# Supplementary material for: Prognostic Value of Troponin Elevation in COVID-19 Hospitalized Patients
Source: J Clin Med. 2020 Dec 17;9(12):4078. doi: 10.3390/jcm9124078 (PMC7766903; doi:10.3390/jcm9124078)
Supplement: Supplementary file 1 [file jcm-09-04078-s001.pdf]

Table S1. Univariate and multivariate analysis of baseline risk factors for death on sensitivity analysis with multiple imputation for missing hsTnI measurements (*n* = 772).

**Risk factor**	**Unadjusted OR (95% CI)**	***p*-value**	**Adjusted OR (95% CI)**	***p*-value**
Age ≥ 65 years old	8.66 (5.26–15.16)	<0.001	4.99 (2.69–9.25)	<0.001
High blood pressure	2.93 (2.01–4.32)	<0.001	0.96 (0.51–1.82)	0.91
Diabetes mellitus	1.57 (1.09–2.26)	0.013	0.85 (0.52–1.37)	0.51
Dyslipidemia	1.82 (1.28–2.57)	<0.001	0.70 (0.43–1.14)	0.15
Tobacco consumption	1.78 (1.19–2.65)	0.004	1.51 (0.88–2.61)	0.13
Active Cancer	3.74 (2.11–6.62)	<0.001	2.52 (1.22–5.22)	0.013
Chronic kidney disease	4.45 (2.93–6.79)	<0.001	1.26 (1.85–5.76)	<0.001
Ischemic heart disease	2.20 (1.37–3.49)	<0.001	0.77 (0.39–1.52)	0.46
Chronic heart failure	3.63 (1.93–6.80)	<0.001	1.66 (0.68–4.03)	0.26
Previous antithrombotic drug	3.50 (2.46–4.99)	<0.001	1.51 (0.86–2.65)	0.15
Previous RASi	1.79 (1.27–2.53)	<0.001	1.14 (0.67–1.92)	0.61
Lymphopenia	2.74 (1.73–4.50)	<0.001	1.80 (0.96–3.37)	0.06
CRP≥100mg/L (max)	1.41 (1.00–2.02)	0.044	2.34 (1.28–4.28)	0.008
Ddimer count (max) ≥3000 µg/L	2.84 (1.67–4.96)	<0.001	1.95 (1.08–3.52)	0.037
hsTroponin elevation	6.63 (3.98–11.25)	<0.001	3.84 (1.78–8.28)	0.008

CI: confidence interval; CRP: C-reactive protein; hs: high-sensitivity; max: maximum; OR: odds ratio; RASi: renin-angiotensin system inhibitor
